# Mississippi Valley Association of Dental Surgeons

**Published:** 1846-09

**Authors:** 


*Mississippi Valley Association of Dental Surgeons.*—The third annual meeting of this association, held at Cincinnati, Ohio, in August, was, we are happy to learn, very well attended, and we expect to have the proceedings, together with the opening address, in time for the next number of the Journal. The importance of union of effort for the advancement of this department of medicine, is now beginning to be felt, as is evidenced by the fact that, since the organization of the American Society of Dental Surgeons, three local associations of dentists have been formed. By this means, the more respectable of the members of the profession are annually brought together, and the improvements and discoveries of all are made known to each. Besides, it affords the different members of the profession an opportunity of becoming acquainted with each other, and of cultivating friendships which would not otherwise have been formed.

We hope that similar associations will soon be organized in all the states of the union.—*Balt. Ed.*

